# *Oceanobacillus aidingensis* sp. nov., a moderately halophilic bacterium

**DOI:** 10.1007/s10482-014-0128-1

**Published:** 2014-03-05

**Authors:** Wenyan Liu, Su Sheng Yang

**Affiliations:** 1 National Engineering Laboratory of Biohydrometallurgy, General Research Institute for Nonferrous Metals, Beijing, 100088 People’s Republic of China; 2 Department of Microbiology and Immunology, College of Biological Sciences, China Agricultural University, Key Laboratory for Agro-Microbial Resource and Application, Ministry of Agriculture, Beijing, 100193 People’s Republic of China

**Keywords:** *Oceanobacillus aidingensis* sp. nov, Moderate halophile, Gram-positive, Salt lake

## Abstract

Two Gram-positive, rod-shaped moderately halophilic bacterial strains, designated AD7-25^T^ and AB-11, were isolated from Aiding and Manasi salt lakes in Xinjiang of China, respectively. The strains were found to be able to grow at NaCl concentrations of 0–21 % (w/v), with optimum growth occurring at 6–8 % (w/v) NaCl. The optimal temperature and pH for growth were determined to be 33–37 °C and pH 7.0–7.5. Cells of the strains are motile by means of polar flagella. Both strains can produce ellipsoidal spores. The major cellular fatty acids were identified as anteiso-C_15:0_, iso-C_15:0_, iso-C_14:0_, anteiso-C_17:0_ and iso-C_16:0_. The diamino acid in the peptidoglycan and the major quinone system were determined to be meso-diaminopimelic acid (meso-DAP) and MK-7, respectively. The DNA G+C contents of stains AD7-25^T^ and AB-11 were 39.8 and 40.0 mol%, respectively. Phylogenetic analysis based on 16S rRNA gene sequences revealed that these two novel strains are closely related to the genus *Oceanobacillus* showing 90–99.5 % similarity with respect to type strains. These two novel strains were most closely related to *Oceanobacillus oncorhynchi* subsp. *incaldanensis* DSM 16557^T^ (99.1 and 99.5 %), followed by *O. oncorhynchi* subsp. *oncorhynchi* JCM 12661^T^ (99.1 and 99.4 %), *Oceanobacillus neutriphilus* CGMCC 1.7693^T^ (97.0 and 97.5 %), *Oceanobacillus sojae* JCM 15792^T^ (97.6 and 98.0 %) and *Oceanobacillus locisalsi* KCTC 13253^T^ (96.5 and 96.9 %). The DNA–DNA hybridization data indicated that DNA relatedness between strains AD7-25^T^ and AB-11 was 91.0 %, and the genomic homology of representative strain AD7-25^T^ with *O. oncorhynchi* subsp. *incaldanensis* DSM 16557^T^, *O. oncorhynchi* subsp. *oncorhynchi* JCM 12661^T^, *O. neutriphilus* CGMCC 1.7693^T^, *O. sojae* JCM 15792^T^ and *O. locisalsi* KCTC 13253^T^ were 41, 39, 20, 23 and 17 %, respectively. On the basis of phenotypic and phylogenetic distinctiveness, strains AD7-25^T^ and AB-11 should be assigned to the genus *Oceanobacillus* as a new species, for which the name *Oceanobacillus aidingensis* sp. nov. was proposed. The type strain is AD7-25^T^ (=CGMCC 1.9106 ^T^ = NBRC 105904^T^).

**Electronic supplementary material:**

The online version of this article (doi:10.1007/s10482-014-0128-1) contains supplementary material, which is available to authorized users.

## Introduction

Molecular and chemotaxonomic analyses have shown that the Gram-positive, rod-shaped and spore-forming moderately halophilic bacteria, which have been isolated from various saline or hypersaline environments (Ventosa et al. 1998; Kim et al. 2007), form several phylogenetically distinct lineages within the family *Bacillaceae*. Many new genera of this kind of bacteria have been reported along with more research ongoing. The genus *Oceanobacillus* was first described by Lu et al. (2001) with the type species *Oceanobacillus iheyensis* DSM 14371^T^ isolated from a deep-sea mud sample. The genus description was later amended due to the isolation of *Oceanobacillus oncorhynchi* subsp. *oncorhynchi* JCM 12661^T^ from the skin of a rainbow trout (Yumoto et al. 2005) and the reclassification of *Virgibacillus* *picturae* (Heyrman et al. 2003) as*Oceanobacillus picturae* DSM 14867^T^ (Lee et al. 2006) isolated from samples of biofilm formation on mural paintings. At the time of writing, twelve *Oceanobacillus* species including two subspecies are recognized. In addition to the three species mentioned above, *O. oncorhynchi* subsp. *incaldanensis* DSM 16557^T^ was isolated from an algal mat collected from a sulfurous spring (Romano et al. 2006), *O. profundus* DSM 18246^T^ from deep-sea sediment (Kim et al. 2007), *O. chironomi* DSM 18262^T^ from a chironomid egg mass (Raats and Halpern 2007), *O. caeni* KCTC 13061^T^ from activated sludge of a wastewater treatment system (Nam et al. 2008), *O. kapialis* KCTC 13177^T^ from fermented shrimp paste (Namwong et al. 2009), *O. neutriphilus* CGMCC 1.7693^T^ from activated sludge of a bioreactor (Yang et al. 2010), *O. sojae* JCM15792^T^ from the bottom of a mold fermenter from the process of soy sauce production (Tominaga et al. 2009), *O. locisalsi* KCTC 13253^T^ from a marine solar saltern (Lee et al. 2010), *O*. *indicireducens* JCM 17251^T^ (Hirota et al. 2013a) from a fermented Polygonum Indigo liquor sample, *O. chungangensis* KCTC 33035^T^ from a sand dune (Lee et al. 2013) and *O. polygoni*  JCM 17252^T^ from a fermented Polygonum indigo (Hirota et al. 2013a, b). The main characteristics of the genus *Oceanobacillus* are as follows: Gram-positive, motile and rod-shaped bacteria, facultatively or obligatory and extremely halotolerant or halophilic. They are also catalase positive but oxidase variable. Voges–Proskauer reaction, indole and H_2_S production and use of citrate are negative. The major cellular fatty acids are anteiso-C_15:0_ and iso-C_15:0_. The main menaquinone type is MK-7. The DNA G+C content range is 35.8–40.1 mol%. The aim of the present study was to elucidate the taxonomic position, using a polyphasic taxonomic approach, of two Gram-positive, rod-shaped moderately halophilic bacterial strains, designated AD7-25^T^ and AB-11, isolated from Aiding and Manasi salt lakes in Xinjiang of China, respectively.

## Materials and methods

### Isolation, morphological and physiological characterization

Two strains, AD7-25^T^ and AB-11, were isolated from sediments of Aiding Salt Lake (89°10′32″–89°54′32″E 42°32′10″–42°49′13″N,127.8 g/l Na^+^, 0.5 g/l K^+^, 0.1 g/lCa^2+^, 0.6 g/l Mg^2+^, 177.5 g/l Cl^−^) and Manasi salt lake (85°37′3″–86°16′20″E 45°37′50″–45°5′47″N,52.4 g/l Na^+^, 11.3 g/l K^+^, 44.6 g/l Mg^2+^, 192.9 g/l Cl^−^) of Xinjiang in China, respectively. For isolation, the samples were suspended in sterilized water supplied with 2 % (w/v) NaCl, serially diluted, spread on improved Gibbson medium (Xu et al. 1995; also used for maintenance), which contained (per litre): 5 g tryptone, 10 g yeast extract, 5 g casein, 2 g KCl, 20 g MgSO_4_·7H_2_O, 20 g NaCl, 3 g trisodium citrate, and then adjusted to pH 7.4. *O. oncorhynchi* subsp*. incaldanensis* DSM 16557^T^, *O. oncorhynchi* subsp. *oncorhynchi* JCM 12661^T^, *O. chironomi* DSM 18262^T^ and *O. neutriphilus* CGMCC 1.7693^T^ were obtained from the culture collections indicated and used as controls in the phenotypic tests.

Based on the proposed minimal standards for the description of aerobic, endospore-forming bacteria (Logan et al. 2009), standard tests were performed for phenotypic characterisation of strains AD7-25^T^ and AB-11. Cell morphology was detected after cultivation in improved Gibbson medium for 16 h. The endospores were detected after 48 h by using a phase-contrast optical microscope (ECLIPSE 50i, Nikon). Transmission electron microscopy (JEM 1230) was used for observing bacterial flagellation. Gram reaction was determined according to the methodology used by Gregersen (1978) and the result was confirmed by the methods described by Doetsch (1981). The presence of endospores was investigated by using the Schaeffer–Fulton staining method (Murray et al. 1994). Colony morphology was examined after 3 days incubation at 33 °C on improved Gibbson medium. The growth conditions such as pH range, NaCl concentrations and temperature range were estimated in improved Gibbson medium by monitoring the increase in optical density at 600 nm. Growth was detected over the temperature range of 4–55 °C (4 and 5–55 °C using increments of 5 °C) by incubating under conditions of 6 % NaCl (w/v) and pH 7.2. Growth at various NaCl concentrations was tested in the range of 0–25 % (w/v) NaCl [0, 0.1, 0.2, 0.3, 0.4, 0.5 % (w/v) and 1–25 % (w/v), using increments of 1 %] by incubating under conditions of 33 °C and pH 7.2. The pH values for growth were tested in the range of 5.0–11.0 (using increments of 0.5 pH units) by incubating under conditions of 33 °C and 6 % NaCl (w/v). For pH experiments, the buffers described by Chen et al. (2007) were used. Growth under anaerobic conditions was determined after incubation in a CO_2_ incubator on anaerobically prepared maintenance medium. Oxygen requirement, activities of catalase, urease and oxidase, hydrolysis of casein, starch, tyrosine, Tween 80, aesculin, xanthine and hypoxanthine, nitrate reduction, Voges–Proskauer test, methyl red test, indole and H_2_S production were performed according to the conventional methods described by Cowan and Steel (1965), Lanyi (1987), Smibert and Krieg (1994) and Dong and Cai (2001). Antibiotic susceptibility was determined on a maintenance medium plate with the following concentrations (μg/ml): rifampicin (5, 10), streptomycin (50, 100), ampicillin (30, 50), gentamicin (15, 40), chloramphenicol (10, 20), kanamycin (50, 100), erythromycin (30, 50), tetracycline (10, 20), spectinomycin (20, 50) and nalidixic acid (15, 30). Fermentation of 49 carbohydrates, arginine dihydrolase, tryptophan deaminase, phenylalanine deaminase, lysine decarboxylases, ornithine decarboxylases, citrate utilization and hydrolysis of gelatin were determined by using API 20E and API 50CHB microtest galleries systems (bioMérieux). Reference strains were used as controls in the tests. All tests were performed in test tubes (3 tubes in parallel) and the tests without inoculation were used as negative controls. Unless otherwise indicated, each test was carried out under the optimal conditions for each strain.

### Chemotaxonomic characterization

Biomass of strains AD7-25^T^ and AB-11 for chemotaxonomic analysis was harvested from cultures after incubation on improved Gibbson medium at 33 °C for 18 h. The analysis of the cell-wall peptidoglycan was carried out with *O. neutriphilus* CGMCC 1.7693^T^ as a reference according to the method described by Schleifer and Kandler (1972) and Schleifer (1985). Cell-wall hydrolysates were separated by one-dimensional chromatography on micro-cellulose thin layers. Menaquinones were analyzed as described previously (Collins 1985) using reverse-phase HPLC (Agilent HPLC-1200). Extraction and analysis of polar lipids by two-dimensional TLC was performed according to Ventosa et al. (1993). Cellular fatty acids were extracted and methylated according to the standard protocol of Sherlock Microbial Identification System version 6.0 (MIDI), analysed by GC (model 6890; Agilent) and identified using the TSBA6 database of the Microbial Identification System (Sasser 1990). The physiological age at the point of harvest of the three bacterial strains tested was the logarithmic growth phase. *O. oncorhynchi* subsp. *oncorhynchi* JCM 12661^T^ was used a reference in chemotaxonomic analysis tests.

### Molecular characterization

Chromosomal DNA was extracted and purified according to standard methods (Marmur 1961). The 16S rRNA gene sequences were amplified as described by Duckworth et al. (1996). PCR products were cloned into the pGEM-T easy vector (Promega) and then sequenced with a DNA Sequencer (373A; Applied Biosystems) and analysed by using the software provided by the manufacturer. The almost-complete 16S rRNA gene sequences of strains AD7-25^T^ and AB-11 (1,464 and 1,468 bp) were compared with sequences from the GenBank, EMBL, DDBJ and PDB databases using the BLAST program via the National Center for Biotechnology Information (NCBI). Pairwise sequence similarities were calculated using the BioEdit software package (Hall 1999). Sequence data were aligned with CLUSTAL W (Thompson et al. 1994). Phylogenetic trees were constructed by the neighbour-joining (Saitou and Nei 1987), minimum-evolution *(*Rzhetsky and Nei 1992 *)* and maximum-parsimony methods (Fitch 1971) with the MEGA5 program package (Tamura et al. 2011). Evolutionary distances were calculated according to the algorithm of the Kimura two-parameter model (Kimura 1980) for the neighbour-joining and minimum evolution methods. Bootstrap analysis was used to assess the stability of the relationships by means of 1,000 resamples. The 16S rRNA gene sequences used for the phylogenetic comparisons are shown in the neighbour-joining phylogenetic tree with their strain designations and accession numbers.

### DNA G+C content and DNA–DNA hybridization

The determination of DNA G+C content was carried out by the thermal denaturation method using a BIO-20 UV spectrophotometer according to Marmur and Doty (1962), and *Escherichia coli* K-12 was used as a standard. Levels of DNA–DNA relatedness were performed by the thermal denaturation and renaturation method described by De Ley et al. (1970) and modified by Huß et al. (1983). DNAs were sheared by sonication (Braun Melsungen) at 50 W for three periods of 10 s. The renaturation was performed in 2 × saline-sodium citrate buffer at 68 °C. In total, three replicate hybridizations were carried out.

## Results and discussion

**Fig. 1:**
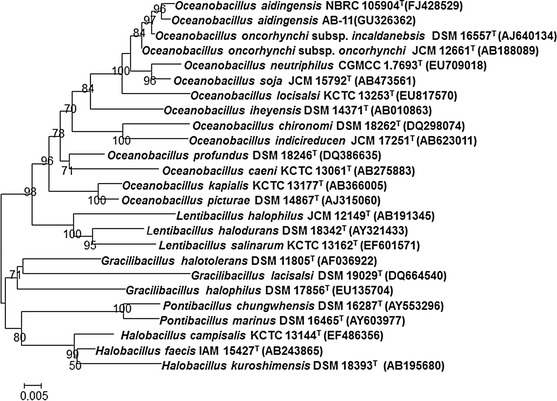
Phylogenetic tree based on 16S rRNA gene sequences and reconstructed using the neighbour-joining method showing the relationship between the isolates, type strains of *Oceanobacillus* and species of other closely related genera. Bootstrap values over 50 % are shown at nodes as percentages of 1,000 replications. *Bar* 0.005 substitutions per site

Two Gram-positive moderately halophilic bacterial strains, designated AD7-25^T^ and AB-11, were isolated from salt lakes in Xinjiang of China. The similarity of 16S rRNA gene sequences (GenBank/EMBL/DDBJ accession numbers FJ428529 and GU326362 respectively) between the two novel strains was 99.1 %. The 16S rRNA gene sequence comparison indicated that the two strains showed 90–99.5 % similarity with respect to the sequences of type strains of validly named *Oceanobacillus* species. Strains AD7-25^T^ and AB-11 were found to be most closely related to *O. oncorhynchi* subsp. *incaldanensis* DSM 16557^T^ (99.1 and 99.5 % sequence similarity), followed by *O. oncorhynchi* subsp. *oncorhynchi* JCM 12661^T^ (99.1 and 99.4 %), *O. neutriphilus* CGMCC 1.7693^T^ (97.0 and 97.5 %), *O. sojae* JCM 15792^T^ (97.6 and 98.0 %) and *O. locisalsi* KCTC 13253^T^ (96.5 and 96.9 %). The sequence similarities with the other *Oceanobacillus* species were 90–95.2 %. Phylogenetic analysis revealed that strains AD7-25^T^ and AB-11 were on the same phylogenetic branch within the genus *Oceanobacillus* (Fig. 1). The topologies of the phylogenetic trees built using the minimum-evolution and maximum-parsimony methods also supported the conclusion that strains AD7-25^T^ and AB-11 form a stable clade with the type strains of *O. oncorhynchi* subspecies (Supplementary Fig. S1). On the basis of sequence analysis, it is evident that the novel strains should be assigned to the genus *Oceanobacillus*.

The DNA–DNA hybridization data revealed that DNA relatedness between strains AD7-25^T^ and AB-11 was 91 ± 5 %. The representative strain AD7-25^T^ and AB-11 shared 41 ± 9 and 42 ± 10 % similarity with *O. oncorhynchi* subsp. *incaldanensis* DSM 16557^T^, 39 ± 3 and 39 ± 7 % with *O. oncorhynchi* subsp. *oncorhynchi* JCM 12661^T^, 20 ± 4 and 21 ± 4 % with *O. neutriphilus* CGMCC 1.7693^T^, 23 ± 6 and 25 ± 7 % with *O. sojae* JCM 15792^T^ and 17 ± 7 and 22 ± 9 % with *O. locisalsi* KCTC 13253^T^ at the genomic level. The DNA G+C contents of strains AD7-25^T^ and AB-11 were determined to be 39.8 and 40.0 mol%, respectively, which are in the range for the genus *Oceanobacillus*.

Phenotypic analysis showed that cells of the strains AD7-25^T^ and AB-11 are Gram-positive and rod-shaped with a size range of 0.5–0.6 × 1.2–2.0 μm. Ellipsoidal endospores were found be formed and the cells to be motile by means of polar flagella (Supplementary Fig. S2). Growth was determined to occur at 4–45 °C, pH 6–10 and 0–21 % (w/v) NaCl. Catalase, urease and oxidase tests were found to be positive, but β-galactosidase was negative. Tween 80 and aesculin were found to be hydrolyzed. H_2_S and indole were not produced. Nitrate was found to be reduced to nitrite. The Voges–Proskauer test was negative. Acids were found to be produced from d-maltose, d-fructose, d-glucose, d-mannitol, d-galactose, d-lyxose, starch, melibiose and sucrose. The morphological, physiological and biochemical characteristics in detail are given in the species description below. The phenotypic differences between the two novel strains and *Oceanobacillus* species are shown in Table 1.

**Table 1:** Differential phenotypic characteristics of strains AD7-25^T^ (AB-11) and the type strains of some phylogenetically related bacteria

Characteristic	1	2	3	4	5	6	7
NaCl for growth (% w/v)							
Range	0–21	0–21	0–22	0–17	5–20	0–15	0–25
Optimum	6–8	6–8	7	3–5	10	2–3	5–8
pH for growth							
Range	6–10	6–10	9–10	6–9	6.5–9.5	6–10	6–9
Optimum	7.0–7.5	7.0–7.5	9	7.0	9.0	8.5	7–7.5
Acid production from							
d-Arabinose	−	−	−	−	−	−	−
Galactose	+	+	+	+	−	+	−
d-Glucose	+	+	+	+	+	+	+
d-Fructose	+	+	+	+	+	+	−
Mannitol	+	−	−	+	+	−	+
d-Trehalose	−	−	+	+	+	−	−
Hydrolysis of							
Aesculin	+	+	−	+	−	−	+
Nitrate reduction	+	+	+	−	+	+	+
Urease	+	+	−	+	+	−	−
β-Galactosidase	−	−	−	+	+	−	−
DNA G+C (mol%)	39.8	40	38.5	36.3	40.1	38	39.8

The results are based on the same test conditions. Species 1, strains AD7-25^T^; 2, AB-11; 3, *O. oncorhynchi* subsp. *oncorhynchi* JCM 12661^T^; 4, *O. neutriphilus* CGMCC 1.7693^T^; 5, *O. oncorhynchi* subsp. *incaldanensis* DSM 16557^T^; 6, *O. sojae* JCM 15792^T^; 7, *O. locisalsi* KCTC 13253^T^. Symbol: +, positive reaction; −, negative reaction

The menaquinones were determined to be MK-7 (93.3 %), MK-6 (3.7 %) and MK-6(H_2_) (3.0 %), respectively. The diamino acid in the peptidoglycan was identified as meso-diaminopimelic acid (meso-DAP). The polar lipids of strain AD7-25^T^ were found to consist of diphosphatidylglycerol, phosphatidylethanolamine and phosphatidylglycerol (Supplementary Fig. S3). The major cellular fatty acids of strains AD7-25^T^ and AB-11 were identified as anteiso-C_15:0_ (44.5, 46.2 %), iso-C_15:0_ (17.5, 15.6 %), iso-C_14:0_ (15.0, 12.8 %), iso-C_16:0_ (9.2, 10.5 %) and anteiso-C_17:0_ (6.9, 7.4 %), respectively. Detailed information on the cellular fatty acid composition, the difference between strains AD7-25^T^, AB-11 and the reference type strains are provided in Table 2.

**Table 2:** Cellular fatty acid compositions of strains AD7-25^T^, AB-11 and O*ceanobacillus* *oncorhynchi* subsp. *oncorhynchi* JCM 12661^T^

Fatty acid	1	2	3	4	5	6	7
*Saturated*							
C_14:0_	0.2	0.3	0.2	1.4	0.1	0.2	tr
C_15:0_	NA	0.1	0.1	–	0.5	2.1	–
C_16:0_	1.7	1.1	1.7	4.6	2.3	1.9	2.3
C_17:0_	2.1	3.0	1.7	–	0.9	3.4	NA
C_18:0_	NA	NA	NA	2.6	0.1	0.7	1.2
*Unsaturated*							
C_16:1_ ω7*c* alcohol	0.2	0.1	0.1	NA	0.3	0.5	–
*Branched*							
Iso-C_14:0_	15.0	12.8	16.4	3.8	12.1	9.8	1.7
Anteiso-C_14:0_	NA	NA	NA	–	0.4	3.0	NA
Iso-C_15:0_	17.5	15.6	13.1	6.8	12.2	4.4	7.3
Anteiso-C_15:0_	44.5	46.2	28.3	37.7	36.5	44.6	46.2
Iso-C_16:0_	9.2	10.5	21.0	15.8	21.2	13.4	6.7
Iso-C_17:0_	1.8	2.7	3.9	4.0	2.9	1.8	4.0
Anteiso-C_17:0_	6.9	7.4	12.6	18.9	10.3	11.3	30.4

Species: 1, Strain AD7-25^T^ (data from this study); 2, Strain AB-11(data from this study); 3, *O. oncorhynchi* subsp. *oncorhynchi* JCM 12661^T^ (data from this study); 4, *O. neutriphilus* CGMCC 1.7693^T^ (data from Yang et al. 2010); 5, *O. oncorhynchi* subsp. *incaldanensis* DSM 16557^T^ (data from this study); 6, *O. sojae* JCM 15792^T^ (data from this study); 7, *O. locisalsi* KCTC 13253^T^ (data from Lee et al. 2010)

*NA* No data available,* tr* trace amount,* –* not detected

Phylogenetic and chemotaxonomic analyses suggested that strains AD7-25^T^ and AB-11 belong to the genus *Oceanobacillus*. However, the DNA–DNA hybridization, DNA base composition and phylogenetic analysis clearly revealed that the two strains are of the same species but are different from the members of this genus. Furthermore, the two strains can be differentiated from the recognized species according to some phenotypic characteristics, such as enzyme activities, growth conditions, acid production from sugars, hydrolysis reactions and nitrate reduction (Table 1 and Description). As a result, it is considered that the two strains represent a novel species of the genus *Oceanobacillus,* for which the name *Oceanobacillus aidingensis* sp. nov. is proposed. The type strain is AD7-25^T^ (=CGMCC 1.9106 ^T^=NBRC 105904^T^).

### Description of *Oceanobacillus aidingensis* sp. nov

*Oceanobacillus aidingensis* (ai.ding.en’sis. N.L. masc. adj. aidingensis from Aiding Salt Lake, where the type strain was first isolated).

Cells are rod-shaped with a size range of 0.5–0.6 × 1.2–2.0 μm, Gram-positive, occurring singly or in pairs and motile by means of polar flagella. Ellipsoidal endospores are located at a central or subterminal position. Colonies are circular, smooth, entire, cream coloured and 1–3 mm in diameter after incubating 3 days. Growth occurs at 0–21 % (w/v) total salts, and the optimum is 6–8 % (w/v). The optimal temperature range for growth is 33–37 °C, and growth occurs at 4–45 °C. The optimal pH value for growth is 7.0–7.5 and pH range is 6–10. Catalase, urease and oxidase are produced but β-galactosidase, arginine dihydrolase, lysine decarboxylase, tryptophan deaminase and ornithine decarboxylase are absent. Tween 80 and aesculin are hydrolyzed but tyrosine, gelatin, xanthine, hypoxanthine, starch and casein are not hydrolyzed. Nitrate can be reduced to nitrite. Citrate cannot be utilized. H_2_S and indole can’t be produced. Methyl red and Voges–Proskauer reaction (acetoin) are negative. According to the API 50CHB system, acids are produced from d-maltose, d-fructose, d-glucose, d-mannitol, d-galactose, d-lyxose, starch, melibiose and sucrose but not from *N*-acetylglucosamine, raffinose, arbutine, gentiobiose, cellobiose, d-trehalose, amygdalin, d-mannose, glycerol, l-rhamnose, salicin, d-tagatose, erythritol, adonitol, methyl beta-d-xyloside, l-sorbose, dulcitol, methyl alpha-d-mannoside, methyl alpha-d-glucoside, melezitose, glycogen, xylitol, d-turanose, dl-fucose, 2-ketogluconate, 5-ketogluconate, dl-arabinose, d-ribose, dl-xylose, inositol, d-sorbitol, d-adonito, dl-arabitol or inulin. Acid production from lactose and gluconate is variable (the type strain is negative). Susceptible to (μg/ml): rifampicin (5, 10), erythromycin (50), kanamycin (100), chloramphenicol (20) and gentamicin (40). But not susceptible to streptomycin (50, 100), ampicillin (30, 50), gentamicin (10), chloramphenicol (10), kanamycin (50), erythromycin (30), tetracycline (10, 20), spectinomycin (20, 50) and nalidixic acid (10, 30). The diamino acid in murein is meso-DAP and the major menaquinone is MK-7, with minor amounts of MK-6 and MK-6(H_2_). Polar lipids of the type strain consist of diphosphatidylglycerol, phosphatidylethanolamine and phosphatidylglycerol. The major cellular fatty acids (≥1 % of the total) are anteiso-C_15:0_, iso-C_15:0_, iso-C_14:0_, iso-C_16:0_, anteiso-C_17:0_, C_17:0_, iso-C_17:0_ and C_16:0_, respectively. The DNA G+C content of the type strain is 39.8 mol%.

The type strain, AD7-25^T^ (= CGMCC 1.9106^T^ = NBRC 105904^T^), was isolated from the Aiding Salt Lake in Xinjiang, China. The GenBank/EMBL/DDBJ accession number for the 16S rRNA gene sequence of strain AD7-25^T^ is FJ428529.

## Electronic supplementary material

Below is the link to the electronic supplementary material.

Supplementary material 1 (DOCX 121 kb)

Supplementary material 2 (DOCX 644 kb)

Supplementary material 3 (PPTX 88 kb)
