# Supplementary material for: Oceanobacillus aidingensis sp. nov., a moderately halophilic bacterium
Source: Antonie Van Leeuwenhoek. 2014 Mar 5;105(5):801–8. doi: 10.1007/s10482-014-0128-1 (PMC3982209; doi:10.1007/s10482-014-0128-1)
Supplement: Supplementary file 1 — Supplementary material 1 (DOCX 121 kb) [file 10482_2014_128_MOESM1_ESM.docx]

Supplementary Figure S1. Phylogenetic tree based on 16S rRNA gene sequences. Numbers at branching points refer to bootstrap values (1000 resamplings; only values above 50% are shown).

(a) maximum-parsimony tree. Bar, 20 expected changes per 1000 nucleotide positions.

(b) minimum-evolution tree. Bar, 0.01 substitutions per nucleotide position.

**
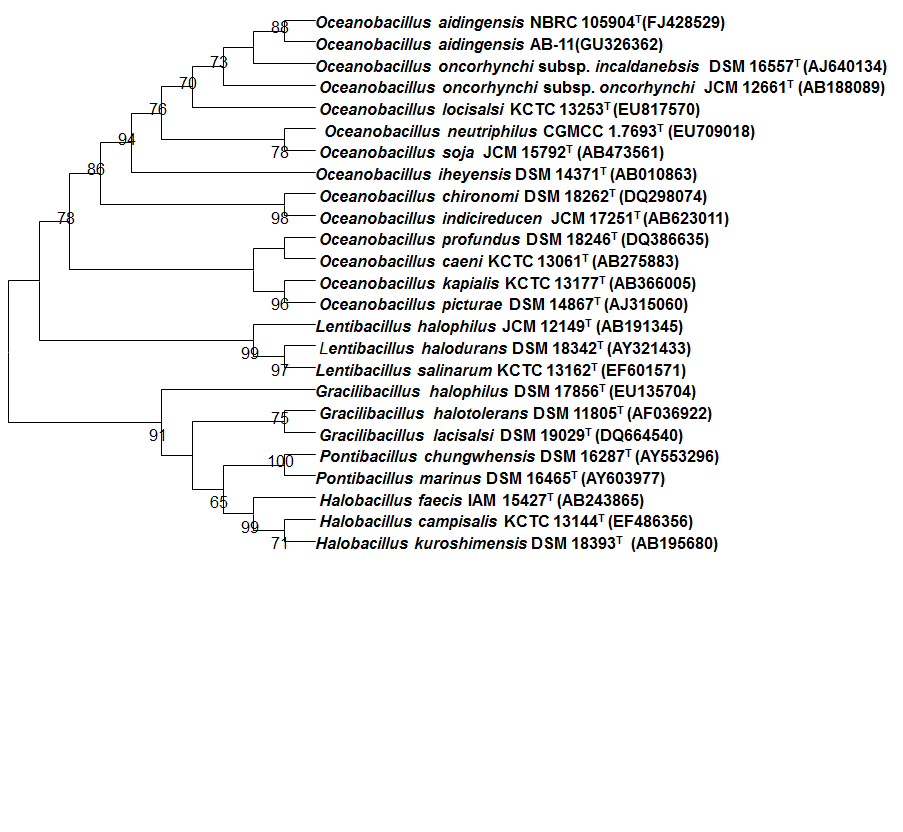
**

**(a)**


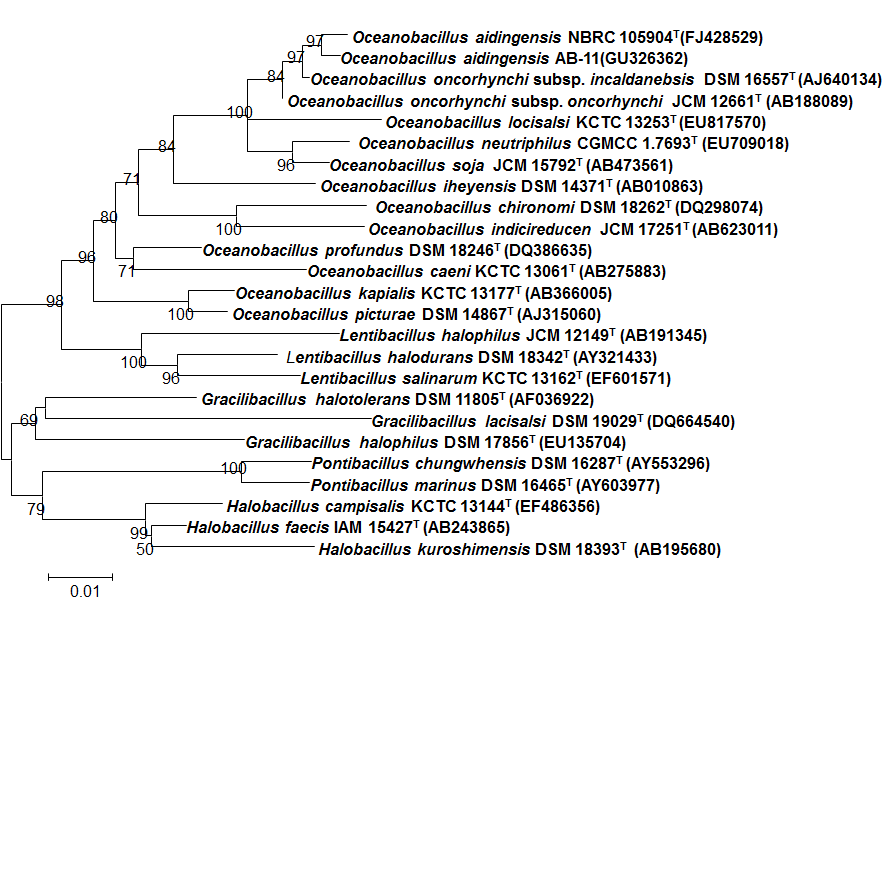


**(b)**
