# Supplementary figures and images for: Oceanobacillus aidingensis sp. nov., a moderately halophilic bacterium
Source: Antonie Van Leeuwenhoek. 2014 Mar 5;105(5):801–8. doi: 10.1007/s10482-014-0128-1 (PMC3982209; doi:10.1007/s10482-014-0128-1)

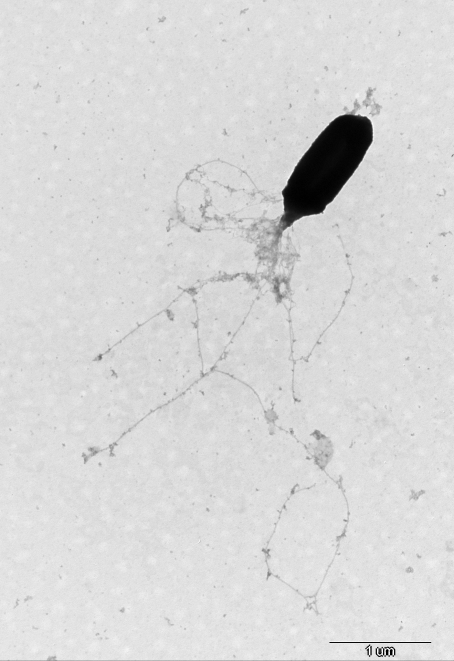

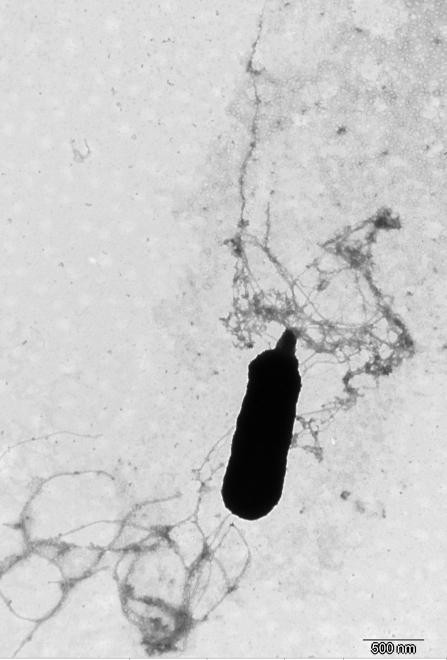


A

B

Supplementary Figure S2 Transmission electron micrograph of AD7-25T. Bar, 1μm (A) and 500nm (B)

Supplement: Supplementary file 2 — Supplementary material 2 (DOCX 644 kb) [file 10482_2014_128_MOESM2_ESM.docx]

## Slide 1
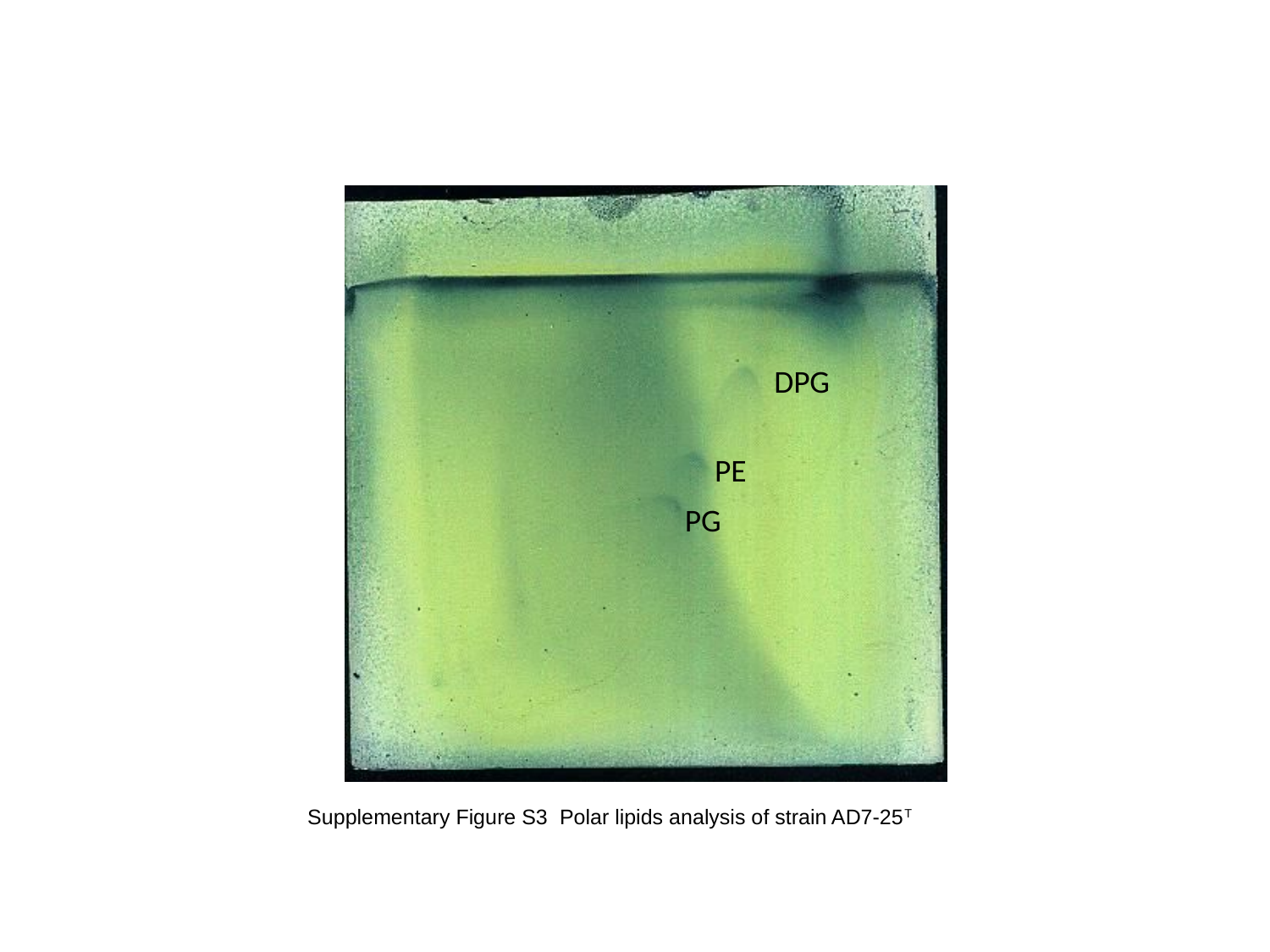

DPG
PE
PG
Supplementary Figure S3 Polar lipids analysis of strain AD7-25T

Supplement: Supplementary file 3 — Supplementary material 3 (PPTX 88 kb) [file 10482_2014_128_MOESM3_ESM.pptx]
